# From Attraction to Repellency: The Olfactory Response Pattern of *Papilio polytes* to Shared Volatiles from Frass and Host Plants Driven by Chemical Composition

**DOI:** 10.3390/insects17050452

**Published:** 2026-04-24

**Authors:** Xue Wu, Zengxin Chen, Yaqi Yang, Huaijian Liao, Yunwei Ju, Chufei Tang

**Affiliations:** 1 College of Forestry and Grassland, Co-Innovation Center for Sustainable Forestry in Southern China, Nanjing Forestry University, Nanjing 210037, China; wuxue034@163.com (X.W.); nlzengxin@163.com (Z.C.); 2 Institute of Leisure Agriculture, Jiangsu Academy of Agricultural Sciences, Nanjing 210014, China; yangyaq0123@163.com (Y.Y.); lhj@jaas.ac.cn (H.L.)

**Keywords:** *Papilio polytes*, behavior, electroantennography, volatile organic compounds

## Abstract

Butterflies locate host sites using plant volatiles, while larval frass cues induce avoidance. This study investigated the olfactory responses of adult *Papilio polytes* to nine shared plant and frass volatiles across 1 × 10^3^ to 1 × 10^6^ ng load doses using electroantennography (EAG) and Y-tube olfactometer assays. EAG responses were significantly influenced by chemical composition, dose, and sex, as well as all their interactions. In contrast, behavioral choices were significantly driven only by chemical composition and its interaction with dose. Although females exhibited higher peripheral sensitivity than males, this physiological sexual dimorphism did not result in significant behavioral differences. Linalool, citronellal, and geraniol were identified as the most potent elicitors. While their single forms and binary blends elicited attraction or neutrality, the ternary mixture significantly repelled both sexes. These results demonstrate that VOC blend composition determines the direction of behavioral responses, with linalool playing a pivotal role in the transition from attraction to avoidance. These findings highlight the importance of shared plant–frass VOCs in host selection by oligophagous insects and provide a foundation for manipulating *P. polytes* behavior.

## Simple Summary

This study investigated how adult common Mormon butterflies (*Papilio polytes*) evaluate host suitability by distinguishing between odors from host plants and caterpillar frass. We identified nine volatile compounds shared by both sources and evaluated their effects using electrophysiological and behavioral assays. Our results show that while individual compounds and binary mixtures are generally attractive or neutral, the ternary blend of the three most potent elicitors (linalool, citronellal, and geraniol) induces strong repellency. This suggests that increasing chemical complexity acts as a warning signal of conspecific competition. Notably, linalool serves as a key mediator in this behavioral shift. These findings enhance our understanding of host selection mechanisms in butterflies and may inform strategies for behavioral manipulation and population management.

## 1. Introduction

Although vision, audition, and other sensory modalities contribute to environmental perception, olfaction constitutes a core sensory foundation that enables insects to adapt to their surroundings and execute essential survival and reproductive behaviors [1,2]. Through olfactory receptors located on their antennae, insects detect volatile organic compounds (VOCs) in the air, transduce these chemical signals into electrophysiological impulses, and transmit them to the central nervous system for integrated analysis of the dynamic environmental information [3,4]. This chemosensory system precisely regulates key behaviors critical for survival and reproduction, including foraging, mate recognition, predator avoidance, and host selection [5].

For phytophagous insects, environmental VOCs serve as information cues utilized by both sexes [6]. Males and females both employ sex pheromones or aggregation pheromones to identify competitors and potential mates during courtship, thereby facilitating the localization and selection of reproductive partners [7,8,9]. Both sexes also rely on plant-emitted VOCs, such as green leaf volatiles and floral nectar aromas, to evaluate the accessibility of food resources, supporting individual survival and energy replenishment [10].

Female phytophagous insects engage in complex reproductive decision-making processes that build upon these foundational chemosensory functions. Through olfactory cues, females assess three critical attributes of host plants: (i) Availability (presence of plant species suitable for larval feeding), (ii) Nutritional quality (plant freshness, developmental stage, and nutrient composition), and (iii) Competition risk (indicators of conspecific/heterospecific larval aggregation inferred from frass-derived VOCs) [5,11,12]. By integrating these multifaceted olfactory signals, females select optimal oviposition sites, thereby providing high-quality food resources for their mobility-limited offspring and ensuring subsequent survival and developmental success. For oligophagous insects that complete development on multiple plant families or genera, the olfactory system of females typically detect evolutionarily conserved chemical signatures shared across different host plants. These signatures generally comprise the “odor profile” of VOC blends (complex mixtures of multiple chemicals) rather than relying on individual chemical components [5,7].

Notably, frass generated through larval feeding serves as a critical ecological information source that further amplifies the complexity of olfactory decision-making in oligophagous insect females. Frass comprises not only undigested plant residue but also metabolites produced by the larval gut microbiota metabolizing host plant substrates, along with partially absorbed plant components remaining from digestion [13,14]. The chemical composition of frass volatiles exhibits strong associations with the host plants, with certain components of the frass being also present in host plant volatiles [9,15,16,17]. The doses, proportional ratios, and combinatorial patterns of these shared volatiles shift dynamically along a gradient corresponding to larval feeding density, therefore signaling “host presence”, “partial resource depletion” and “intensified intraspecific competition” [5,18]. For female insects, analyzing and responding to signals emitted jointly by host plants and frass volatiles is essential for accurately assessing overall oviposition site suitability [19,20]. Consequently, understanding this response pattern is crucial for unraveling how female oligophagous insects synthesize conflicting olfactory cues, negative (e.g., frass volatiles indicating competition) versus positive (e.g., host plants volatiles indicating food resources), into a coherent oviposition strategy. Yet, research has largely focused on the isolated effects of either host or frass volatiles, leaving the integrative role of their shared components in behavioral guidance underexplored.

*Papilio polytes* Linnaeus (the common Mormon butterfly) is an oligophagous species geographically distributed from tropical to northern temperate zones. Its larvae develop on multiple host plants within the Rutaceae family, including key genera such as *Citrus* spp. and *Zanthoxylum* spp., while its flower-visiting adults contribute to pollination across diverse plant species [21,22,23]. Although sometimes perceived as a potential pest in citrus cultivation regions, its ecological role as a pollinator has been validated by multiple studies, underscoring its dual impact on both agriculture and ecosystem services [21,24]. Meanwhile, this species exhibits remarkable research and economic values: Larvae display various mimicry strategies, and adults show remarkable polymorphism in wing coloration patterns, making the species not only a model system for biological research but also a flagship species in the ornamental butterfly industry [25,26]. Elucidating its oviposition preferences guided by VOC recognition will not only yield technical solutions for its oviposition regulation in scaled breeding systems and field population management, but also provide foundational insights into the olfaction-mediated decision-making mechanisms that drive oviposition in oligophagous insects [27,28].

This study uses *P. polytes* as a model organism to investigate the olfactory response patterns to VOCs shared between frass and host plants of oligophagous insects. Although females may have evolved more acute chemical senses due to their egg-laying needs, given that males also need to use plant signals to find food or mates, this study simultaneously examined the response patterns of both sexes to explore the conservation of this chemical recognition mechanism. We first identified shared VOCs through GC MS analysis and a comprehensive literature review. We then employed EAG to quantify neural responses of both sexes across a dose gradient of 1 × 10^3^ to 1 × 10^6^ ng. Building on these results, Y-tube olfactometer assays were conducted to examine how chemical composition and dose interplay to modulate behavioral responses in both sexes. Data were analyzed using generalized linear models in R to assess the effects of multiple factors and their interactions. By integrating chemical data, electrophysiological measurements, and behavioral observations, we systematically explored the olfactory response patterns of *P. polytes* to shared VOCs.

## 2. Materials and Methods

### 2.1. Materials

#### 2.1.1. Insects

All samples of *P. polytes* were laboratory-reared under identical microclimatic conditions (>5 generations at 25.0 ± 1.0 °C, 75 ± 5% RH, 14:10 L:D) at the Institute of Leisure Agriculture, Jiangsu Academy of Agricultural Sciences, representing a long-term experimental population. Larvae were fed exclusively with fresh lemon (*Citrus limon* (Linnaeus)) leaves. All leaves were of uniform size and coloration, collected from 8-year-old lemon plants cultivated under uniform environmental conditions and verified to be free from pests and diseases. For the electroantennography (EAG) and behavioral (Y-tube olfactometer) response experiments, adult butterflies of both sexes, identified based on morphological characteristics, were selected at 2 days post-eclosion. All selected individuals were unfed and exhibited normal wing expansion and flight capability.

#### 2.1.2. Frass

Frass samples were derived from 3rd–5th instar larvae of *P. polytes* reared under the aforementioned standardized conditions (>5 generations at 25.0 ± 1.0 °C, 75 ± 5% RH, 14:10 L:D). This larval stage was chosen to efficiently obtain sufficient frass mass within a short timeframe, thereby minimizing temporal effects on frass volatiles. Freshly excreted larval frass was collected daily at a fixed time from the bottom of the plastic rearing box, where it had fallen through the gaps between the support twigs placed inside to aid climbing and leaf placement for the larvae. The frass was immediately sealed, and stored in a 4 °C refrigerator and subjected to volatile compound extraction on the third day after collection. All frass samples originated from a single cohort of larvae to minimize inter-individual variability.

#### 2.1.3. Volatiles

To identify volatiles shared between larval frass and host plants, we first analyzed the frass volatile profile using GC-MS and screened for major constituents with a relative abundance greater than 0.5%. Host plant volatiles were identified through literature review, focusing primarily on the volatile constituents present in the leaves not only of *C. limon* (used to insect feeding), but also other common host plants belonging to the *Citrus* and/or *Zanthoxylum* genera [29,30,31,32,33,34]. The two profiles were then compared to identify the key VOCs common to both sources. For subsequent EAG and Y-tube olfactometer assays, we used analytical-grade standards of these shared compounds to ensure precision and comparability.

### 2.2. Methods

#### 2.2.1. Profiling the VOCs of Larval Frass

Headspace solid-phase microextraction coupled with gas chromatography–mass spectrometry (HS-SPME-GC-MS) was employed to identify the chemical composition and relative abundance of volatile compounds in *P. polytes* frass. For each test, one gram of larval frass sample was placed in a 20 mL headspace vial, followed by the addition of approximately 5 mL of saturated sodium chloride solution to enhance volatile release. The vial was immediately sealed. The headspace vial was placed in an 80 °C water bath for 20 min to allow sufficient volatile compounds partitioning into the headspace.

A 50/30 μm divinylbenzene/carboxen/polydimethylsiloxane (DVB/CAR/PDMS) solid phase microextraction (SPME) fiber (Supelco 57348-U, Bellefonte, PA, USA) was used for VOC extraction. This fiber coating was chosen for its broad affinity for volatile and semi-volatile compounds across a range of polarities. Prior to first use, the fiber was conditioned in the GC-MS injector port at 250 °C for 60 min under helium flow. Between samples, the fiber was reconditioned at 250 °C for 10 min to eliminate carryover. Blank runs were performed daily to verify fiber cleanliness.

The sealed vial was immersed in a thermostatted water bath at 80.0 ± 0.5 °C and equilibrated for exactly 20.0 min to allow VOCs to partition into the headspace. After equilibration, the conditioned SPME fiber was exposed to the headspace (needle penetration depth: 22 mm) and maintained at 80 °C for 30.0 min under static conditions. Following extraction, the fiber was immediately retracted and inserted into the GC-MS injector (250 °C) for thermal desorption in splitless mode for 5.0 min. Three independent replicates were performed for each sample.

Analyses were performed using an Agilent 5975B GC-MS system equipped with an HP-5MS capillary column (30 m × 0.25 mm i.d., 0.25 μm film thickness; Agilent J&W, Folsom, CA, USA). Helium (99.999% purity) was used as carrier gas at a constant flow rate of 1.0 mL/min. The oven temperature program was: initial 50 °C held for 2 min, increased at 5 °C/min to 180 °C and held for 5 min, then increased at 10 °C/min to 250 °C and held for 5 min (total run time: 46 min). The injector temperature was 250 °C, and the transfer line was held at 280 °C. The ion source (EI, 70 eV) and quadrupole temperatures were set to 230 °C and 150 °C, respectively. Mass spectra were acquired in full scan mode over a range of m/z 40–600.

Chromatographic peaks were identified by comparing their mass spectra with the NIST 2020 mass spectral database (similarity ≥ 80%) and by matching experimentally determined linear retention indices with those of authentic standards or literature values. Retention indices were calculated using a C8–C20 n-alkane series (Sigma-Aldrich, Darmstadt, Germany) analyzed under identical GC conditions. Relative abundances of individual VOCs were expressed as percentages of the peak area of each compound relative to the total peak area of all detected compounds (excluding column bleed, siloxane artifacts, and background ions present in blank runs). Only compounds with a signal-to-noise ratio ≥ 3 and detected in all three replicates were considered for reporting.

To verify whether the 30 min extraction time was sufficient for comparative profiling, after the first extraction and GC-MS analysis, the same vial was immediately subjected to a second identical extraction using a reconditioned SPME fiber. The total peak area of the second extraction accounted for less than 5% of that of the first extraction for all detected compounds, and the majority of individual compounds fell below the signal-to-noise threshold of 3. These results indicate that a single 30 min extraction captures the vast majority of extractable VOCs under standardized conditions, and that the relative abundances obtained are reliable for comparative purposes.

#### 2.2.2. Preparation of Volatile Solutions for EAG and Y-Tube Olfactometer Assays

All volatile compounds were dissolved in analytical-grade n-hexane. For single-compound tests, four serial dilutions of each volatile were prepared, corresponding to final concentrations of 0.1, 1, 10, and 100 μg/μL. Following the initial screening, three compounds of particular interest were selected for further mixture studies: (1) the compound eliciting the maximum EAG response, and (2) compounds whose EAG responses reached at least 50% of this maximum value at any given concentration (dose). Three compounds met these criteria: linalool, citronellal, and geraniol. They were then combined to formulate four distinct mixtures: three binary mixtures (equal mass ratios of each pairwise combination) and one ternary mixture (equal mass ratio of all three compounds). Each mixture was subsequently serially diluted in n-hexane to match the same concentration (dose) gradient (0.1, 1, 10, and 100 μg/μL) as that used for the individual compounds, with the concentration referring to the sum amount of all VOCs present in the binary or ternary mixture.

To establish a biologically relevant control and validate the olfactory assay system, a crude extract of larval frass was prepared and tested in parallel. One gram of fresh larval frass was immersed in 10 mL of n-hexane and allowed to macerate at room temperature for 24 h. The resulting supernatant was filtered and serially diluted with n-hexane to produce doses corresponding to the standard dilution series of synthetic volatile solutions. This frass extract served as a critical control to benchmark EAG response and repellent efficacy of individual compounds and their mixtures against the authentic olfactory cue.

#### 2.2.3. EAG Recordings

EAG recordings were conducted following the protocol of Zhu et al. [35] using a Syntech apparatus (Kirchzarten, Germany) equipped with an IDAC-4 signal amplifier (Kirchzarten, Germany) and EAG Pro software (version v2.02). Prior to dissection, test insects were acclimated for 1 h in a ventilated cage free of irritant odors. Antennae were excised at their bases for the experiment. During the experiment, the apical 0.5 mm of each antenna was removed, and only the middle sections were used. Test stimuli were prepared by applying 10 µL of each solution (single compounds, mixtures, or frass extract at all dose gradients, corresponding to loaded dose gradients of 1, 10, 100 and 1000 μg) onto filter paper strips (0.6 × 2 cm). After allowing the solvent to evaporate for 2 min, the strips were inserted into disposable Pasteur pipettes and sealed with Parafilm until use.

Antennae were mounted between two glass EAG electrodes coated with conductive gel (SignaGel, Parker Laboratories; Fairfield, NJ, USA) and inserted into the EAG probe. Prior to formal testing, antenna viability was assessed by applying a brief air puff and observing the signal response: a clear peak indicated a viable antenna, whereas its absence led to replacement. The baseline was allowed to stabilize before proceeding.

For the formal assay, a 60 s baseline was recorded to ensure signal stability. Stimuli were presented in the following sequence: n-hexane (control), followed by increasing concentrations (doses) of each test compound. Each stimulus lasted for 0.3 s, with a 1 min interval between consecutive stimulations to prevent antennal adaptation. Each antenna was tested with only one compound or mixture. For each concentration gradient of the test solution, 20 antennae (10 from males and 10 from females) were tested. In some cases, antennae for a given sex were sourced from more than five individuals, as certain individuals provided only one antenna. Raw signals were amplified by the IDAC-4 unit and digitized by EAG Pro software.

#### 2.2.4. Behavioral Assay Using a Y-Tube Olfactometer

A custom-built Y-tube olfactometer was used to assess the behavioral responses of *P. polytes* to various volatile compounds. The airflow system was constructed by sequentially connecting the following components to generate a continuous, purified airstream: an atmospheric sampler (QC-1S; Beijing Ke’an Labor Protection New Technology Co., Ltd., Beijing, China), followed by an activated carbon scrubber bottle, a distilled water bubbler, a glass rotameter (LZB-3WB; Xiangjin Flow Instrument Factory, Changzhou, China), and finally the Y-tube chamber (inner diameter: 12 cm; arm length: 30 cm; branching angle: 60°; Beijing Zhuoyuan Hangcheng Technology Development Co., Ltd., Beijing, China). An oil-free vacuum pump (SCI-HS25ZF; Tianjin Shengze Technology Co., Ltd., Tianjin, Chian) pulled air through the entire system. Airflow rate was regulated by the rotameter and maintained at a constant 800 mL/min, corresponding to an air velocity of 0.27 m/s.

Because the induced EAG response intensity at the 1 × 10^3^ ng dose (10 μL × 0.1 μg/μL) was relatively low (with an average value below 0.1 mV), this dose was excluded from the behavioral assays. Y-tube evaluations therefore tested behavioral responses at 1 × 10^4^, 1 × 10^5^, and 1 × 10^6^ ng load mass. For each trial, equal volumes (10 μL) of test solution (corresponding to 1 × 10^4^, 1 × 10^5^, and 1 × 10^6^ ng mass load of the respective compound or blend) and a control solution (n-hexane) were applied to separate filter paper discs. These discs were placed into individual odor-source jars attached to the distal end of each arm of the Y-tube, with treatment and control positions randomly assigned for each replicate to mitigate positional bias.

A single adult butterfly was introduced at the base of the olfactometer. A timer was initiated, and the insect’s movement was observed. A choice was recorded if the butterfly entered an arm and remained there for at least 30 s. If no such commitment occurred within 10 min, the trial was terminated and recorded as “no choice.” Each combination of compound and dose was tested using 25 males and 25 females, resulting in a total of 50 insects tested per treatment, maintaining a 1:1 sex ratio within each testing batch.

To minimize contamination and positional bias, the following procedures were implemented after every five trials: (1) the entire apparatus was thoroughly rinsed with anhydrous ethanol and air-dried; (2) fresh odor-source jars and filter paper discs were prepared; and (3) the left/right positions of the odor sources were switched. Throughout the experiment, ambient light conditions were standardized, with all procedures conducted in complete darkness and observations made via night vision camera to eliminate phototactic effects. Adults were kept under the same photoperiod regime as the larvae (14:10 h L:D; light phase from 06:00 to 20:00). All behavioral tests were performed during the light phase, specifically between the 4th and 11th hours after lights-on (i.e., 10:00 to 18:00). For each trial, the insect was transferred from the standard light conditions into experiment conditions.

#### 2.2.5. Statistical Analysis

All statistical analyses and data visualization were conducted using R (version 4.5.1), primarily with the ggplot2 [36], emmeans [37], and multcomp [38] packages. Throughout the text, statistical significance was set at *p* < 0.05. The specific analytical procedures for each dataset are described below.

For EAG data analysis, we preprocessed the raw data by converting the dose variable (ng) and EAG response variable (mv) to numeric format, and retained the solvent control (dose = 0) as a valid experimental level in the dataset without prior subtraction, allowing the model to directly estimate the response under control conditions. We then employed generalized linear models (GLMs) to analyze the EAG response values (mv) with sex, stimulus (compound or mixture), and dose (ng) as fixed effects, including all two- and three-way interactions. The significance of each term was assessed via analysis of deviance using Type II Wald chi-square (χ^2^) tests via the anova function. Post hoc pairwise comparisons were conducted using estimated marginal means (EMM) with the emmeans package. Specifically, we compared responses between sexes for each compound, responses among compounds within each sex, and responses among doses for each sex and compound combination. Due to significant interactions observed in the model, all post hoc comparisons were performed within the context of the other factors. Results were summarized using compact letter displays (CLDs) to denote statistically homogeneous subgroups.

For Y-tube olfactometer assays, we first tested whether the proportion of insects selecting the treatment arm among all tested individuals differed from the control arm using χ^2^ tests for each compound–dose–sex combination, following the criteria: attractive if treatment selection > 50% and *p* < 0.05; repellent if control selection > 50% and *p* < 0.05. To further investigate the joint effects of compound, sex, and dose on behavioral responses, we employed a GLM with a binomial distribution and logit link function. The model included compound, sex, dose, and all their interactions as fixed effects. Significance of each term was assessed using likelihood ratio tests through analysis of deviance. Post hoc pairwise comparisons among compounds within each sex were conducted using EMM calculated on the logit scale via the emmeans package to resolve differences in behavioral response intensities.

## 3. Results

### 3.1. Identification of Shared Volatiles

HS-SPME-GC-MS analysis of *P. polytes* larval frass revealed a complex mixture of volatile organic compounds. A total of 26 compounds were consistently detected across all three replicates and were therefore included in subsequent analyses (Table 1). These compounds were classified into several chemical categories based on their structural and functional characteristics. Sesquiterpene hydrocarbons constituted the most abundant chemical class, with (E)-β-farnesene (25.84 ± 6.04%) and β-caryophyllene (19.70 ± 3.99%) being the major components, collectively accounting for 45.54% of the total detected volatiles. Monoterpenoids were also prominent, particularly citronellol (7.81 ± 2.12%) and (R)-(+)-citronellal (6.06 ± 3.67%), both of which show characteristic citrus-like aromas [29,30,31,34]. Other notable monoterpenoids included linalool (1.00 ± 0.37%), α-terpineol (1.02 ± 0.12%), and (R)-(+)-Limonene (1.38 ± 0.38%). Aromatic compounds such as 2,3-dihydrobenzofuran (0.38 ± 0.06%), eugenol (0.34 ± 0.09%), and olivetol (0.23 ± 0.04%) were detected in minor quantities. Additionally, oxygenated sesquiterpenoids, including spathulenol (2.13 ± 0.27%), caryophyllene oxide (1.42 ± 0.57%), and (+)-nootkatone (0.41 ± 0.06%), were identified. Trace amounts of lactones (e.g., eldanolide, 0.59 ± 0.10%) and sesquiterpene alcohols (e.g., α-bergamotol, 0.25 ± 0.01%) were also present.

Based on a literature review of the major host plants of *P. polytes*, nine commonly occurring host plant volatiles were selected for bioassays: (R)-(+)-Limonene, linalool, citronellal, β-caryophyllene, citronellol, geraniol, (E)-β-farnesene, β-bisabolene, and caryophyllene oxide. These compounds are characteristic of, or commonly found in, the leaves of *Citrus* and/or *Zanthoxylum* species [29,30,31,32,33,34].

### 3.2. EAG Responses of P. polytes to VOCs

The GLM analysis revealed that all main effects including chemical composition, sex, and dose, as well as their interactions, significantly influenced EAG response magnitudes (Table 2). Overall, females exhibited significantly stronger EAG responses than males across all test conditions, and highly significant differences were also observed among compounds and doses (Supplementary Table S1). Female responses, characterized by the estimated marginal means (EMM), were consistently higher than male responses for all individual and mixed VOCs (Supplementary Table S2).

Significant differences in EAG responses were observed among compounds within each sex (Supplementary Table S3, Supplementary Figures S1 and S2). For females, the linalool + citronellal mixture produced the highest response among all stimuli at 0.298 mV, which exceeded all individual compounds and most mixtures. Among individual compounds, linalool induced the strongest female response at 0.201 mV, followed by geraniol at 0.161 mV and citronellal at 0.121 mV, while beta bisabolene was the weakest at 0.074 mV. For males, the four synthetic mixtures showed comparable responses between 0.188 and 0.205 mV, and these values were all significantly higher than linalool alone at 0.137 mV. Among individual compounds, linalool again induced the highest male response at 0.137 mV, followed by geraniol at 0.112 mV and (E)-β-farnesene at 0.107 mV. In contrast, β-bisabolene and caryophyllene oxide were the weakest stimuli for males at 0.061 and 0.064 mV, respectively. The larval frass extract elicited significantly weaker responses than synthetic mixtures in both sexes.

**Figure 1:**
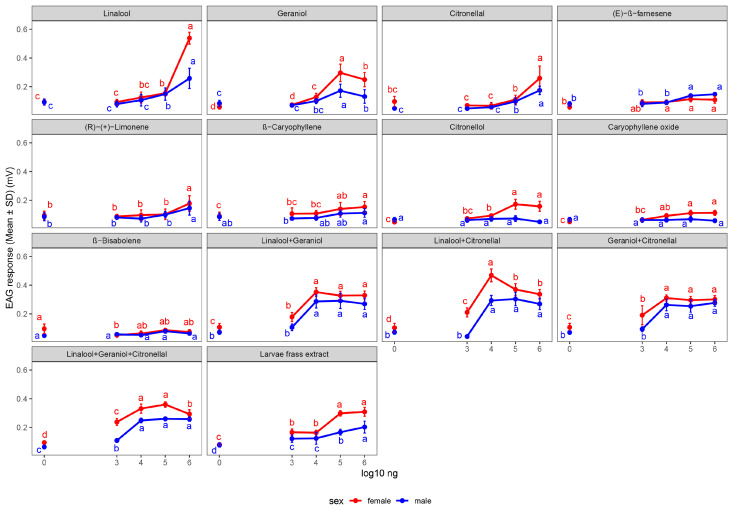
EAG dose–response curves of *P. polytes* to 14 stimuli. Mean ± SD responses of females (red) and males (blue) to nine individual VOCs, four synthetic mixtures, and larval frass extract. Responses are plotted against log_10_-transformed dose (ng). Different lowercase letters above data points indicate significant differences among doses within each sex-stimulus combination (emmeans, Tukey-adjusted *p* < 0.05).

Dose–response analysis (Figure 1) showed that EAG responses increased with dose for most compounds in both sexes, with patterns differing between individual and mixed compounds (Supplementary Table S4). For females, responses at 1 × 10^6^ ng exceeded control levels for all individual compounds except β-bisabolene. Linalool showed the steepest dose–response, with the highest dose (0.538 mV) being 5.7 times the control (0.094 mV). In males, significant dose-dependent increases occurred for linalool, citronellal, geraniol, and (E)-β-farnesene, while caryophyllene oxide, citronellol, and β-bisabolene showed no elevation above control. For mixed compounds, several mixtures peaked at intermediate doses. The linalool + citronellal mixture reached its maximum female response at 1 × 10^4^ ng (0.469 mV), with no further increase at higher doses; the linalool + geraniol mixture plateaued above 1 × 10^4^ ng. In contrast, the larval frass extract exhibited a monotonic dose–response in both sexes, increasing significantly from 1 × 10^3^ to 1 × 10^6^ ng (*p* < 0.001). Female responses to mixed compounds generally peaked at lower doses (1 × 10^3^–1 × 10^4^ ng) than male responses, which often continued to increase up to 1 × 10^6^ ng.

### 3.3. Behavioral Responses of P. polytes to VOCs in Two-Choice Assays

**Figure 2:**
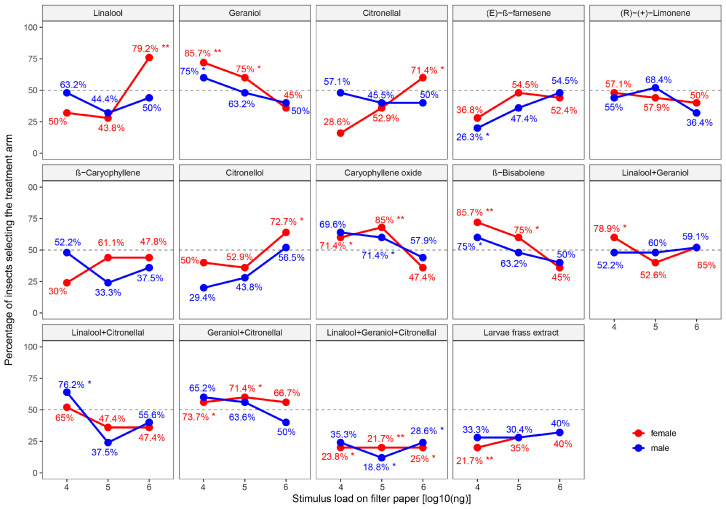
Dose–response curves of *P. polytes* to VOCs in Y-tube olfactometer assays. Each curve shows the percentage of insects that chose the treatment arm among all tested individuals plotted against the log_10_-transformed dose. Data points marked with an asterisk (*) or double asterisks (**) indicate a significant difference in choice percentage between the treatment and the control (n-hexane) for that specific compound–dose–sex combination (χ^2^ test), where * represents *p* < 0.05 and ** represents *p* < 0.01. The number beside each point represents the proportion of selecting individuals among those that made a choice.

Initial χ^2^ tests for each compound–dose–sex combination, based on the percentage of insects that chose the treatment arm among all tested individuals, revealed significant behavioral effects for several compounds (Figure 2). Among the nine individual volatile compounds tested, six elicited significant attraction or repellence, while geraniol, β-caryophyllene, and (R)-(+) limonene showed no significant effect at any tested dose. Linalool, citronellol, and citronellal were significantly attractive to females at the highest dose of 1 × 10^6^ ng. Caryophyllene oxide and β-bisabolene attracted females at intermediate doses of 1 × 10^4^ to 1 × 10^5^ ng. (E)-β-Farnesene repelled males at 1 × 10^4^ ng. No other significant attraction or repellence was detected for individual VOCs. Regarding mixed VOCs, the ternary mixture of linalool, citronellal, and geraniol, as well as the larval frass extract, repelled both sexes across multiple doses. Among binary mixtures, linalool and citronellal attracted males only at 1 × 10^4^ ng, while geraniol mixed with either citronellal or linalool attracted females at specific doses. The proportion of responders that selected the treatment was also calculated. The overall dose–response trends of the two proportions remained largely consistent for all compounds and both sexes, except in treatments such as (R)-(+)-limonene and citronellol. In these cases, similar overall choice percentages between adjacent doses led to slight shifts in the relative order of the two proportions.

The GLM analysis, which was also conducted based on the percentage of insects that chose the treatment arm among all tested individuals, revealed that compound (*p* < 0.001) and the chemical composition × dose interaction (*p* < 0.001) were the only significant predictors of behavioral choice (Table 3). Sex did not exert a significant main effect, nor did it interact significantly with compound or dose, indicating that male and female behavioral responses were statistically comparable across all treatments. The significance of the interaction between composition and dose indicates that different compounds exhibited distinct response patterns across the tested range. This is further resolved by the comparisons of estimated marginal means (EMM) on the logit scale (Supplementary Table S5). For both sexes, the ternary mixture and larval frass extract belonged to the lowest EMM groups, representing the strongest repellent effects observed. In contrast, individual compounds such as geraniol, β-bisabolene, and caryophyllene oxide were positioned in the highest EMM groups, reflecting their relatively higher attractive potential compared to the more complex blends.

The GLM confirmed that sex did not significantly modulate behavioral responses (Table 3). Although sex differences were observed at certain dose points (e.g., the repellent effect of (E)-β-farnesene on males, Figure 2), these local differences did not reach overall significance when included in the global GLM model that accounted for all factors. This indicates that the behavioral recognition pattern of this species is largely consistent between sexes, and that both sexes utilize these shared volatiles in a similar manner to navigate their environment.

## 4. Discussion

This study systematically evaluated the electrophysiological and behavioral responses of adult *P. polytes* to shared volatiles derived from larval frass and host plants. Statistical analyses revealed that chemical composition, dose, and sex, as well as their mutual interactions, all significantly influenced EAG responses. In contrast, behavioral responses were significantly shaped only by chemical composition and its interaction with dose, whereas sex and its associated interactions showed no significant effects. Although females exhibited heightened peripheral sensitivity to the shared volatiles compared to males, this physiological sexual dimorphism did not translate into differences in final behavioral choices. These results provide a critical foundation for understanding the chemical communication between life stages of oligophagous butterflies and the chemical mechanisms underlying their host-location strategies.

In the present study, distinct EAG responses were generally accompanied by clear behavioral attraction or repellency. However, the two measures were not always consistent. For instance, geraniol elicited strong EAG responses in *P. polytes* but did not produce significant attraction or repellency in either sex. Similar discrepancies have been reported in olfactory studies of other insects, including *Trichogramma dendrolimi* and *Trabala vishnou gigantina* [39,40]. This is because EAG primarily measures the initial detection of odors by the antennae. It confirms whether an insect can detect a chemical, but it does not reflect how the insect’s brain processes that information and perceives it. In contrast, behavioral assays show the final “decision” made by the insect. A chemical might trigger a strong physiological signal without being “important” enough to cause the insect to move toward or away from it [41,42,43]. Accordingly, the following discussion focuses primarily on behavioral outcomes to evaluate the attractive and repellent effects of host-plant and frass volatiles on *P. polytes*.

The differences in the responses to different VOCs indicated that linalool may play a significant role in host selection of female *P. polytes*. Among all the single-component groups tested, it not only induced the strongest behavioral responsiveness at the highest dose, but also induced a shift from attraction to repellency when being mixed with other compounds. This inference is supported by previous studies. Linalool has been shown to provoke strong EAG responses while inducing oviposition behavior in female *P. polytes*, and to attract females of *P. maackii*, another citrus-feeding species [32,44]. Moreover, linalool is a major volatile in male wings of *P. polytes* [45], suggesting it may coordinate behaviors spanning mate selection to oviposition decision-making.

The behavioral impact of linalool is highly context-dependent: despite an identical total loading, the response shifted from attraction to repellency when the composition changed from a single compound to a binary mixture and further to a ternary blend, with the ternary blend eliciting steeper response slopes. These findings suggest that volatile complexity is crucial for *P. polytes* to discriminate between host plants and larval frass, shifting the focus from individual compounds to the recognition of entire chemical fingerprints [46]. While host location often relies on specific profiles, the repellent effects of frass are generally associated with more complex mixtures that may serve as a chemical warning signal of high larval density and intensified competition [47,48,49]. Such multilevel neural integration enables precise ecological decision-making within complex environments [50,51]. Consequently, elucidating the synergy between linalool and other volatiles is essential for understanding the cognitive mechanisms that guide the host-selection strategies of this butterfly.

This study also revealed that the interaction between dose and chemical composition acts as a modulator of *P. polytes*’ behavioral responses to volatiles. This interaction highlights that *P. polytes* may possess different response thresholds for mixtures compared to single components. For instance, complex mixtures were effective even at low doses and remained stable across the tested range. In contrast, single compounds like linalool required much higher doses to produce similar effects. Unlike systems such as *Cotesia plutellae*, where females switch from attraction to repellency as the dose of (E)-2-hexenal increases from 10 μg to 1000 μg (equivalent to 1 × 10^4^ to 1 × 10^6^ ng) [52], *P. polytes* displayed consistent behavioral valence within the tested dose range of 10 to 1000 μg. These findings suggest that herbivorous insects do not just respond to dose in isolation because the chemical context determines the optimal dose required to trigger specific ecological behaviors [53,54]. This may also explain why no significant behavioral differences were observed between females and males in this study. Consequently, identifying the precise dose and composition interplay is essential for reliably predicting and inducing the desired attractive or repellent effects in *P. polytes*.

Still, several limitations of this study should be acknowledged. First, the volatile profiles used in our assays were derived from stored frass that had not been exposed to soil or leaves, conditions that differ from those in natural settings. Because volatiles can change over time and be affected by environmental microorganisms, this storage and isolation may have altered the VOC composition [55]. Although larval frass is known to retain oviposition-deterring activity for several days [47,56], the experimental conditions used here may have omitted certain VOCs involved in attraction or avoidance behaviors under more natural conditions. Second, our experiments used synthetic compounds at fixed doses and ratios, whereas natural volatile emissions vary continuously in space and time [57]. Additionally, the assays measured only initial orientation responses and did not assess subsequent behaviors such as landing or oviposition [32]. Finally, the inconsistencies between EAG and behavioral responses remain unresolved. Future research should examine a more ecologically relevant range of lower doses along with varying blend ratios, which would help clarify the neural mechanisms underlying blend recognition and refine the findings presented here.

## 5. Conclusions

Our integrated analysis demonstrates that the olfactory response of *P. polytes* to shared plant and frass volatiles is primarily driven by blend composition and its interaction with dose. While electrophysiological sensitivity is significantly modulated by the interplay of sex, dose, and composition, the actual behavioral decision-making process is sex independent and relies on the integration of chemical context. Linalool, citronellal, and geraniol were the three most potent elicitors, and their ternary combination served as a functional repellent. This transition from attraction to repellency as the blend becomes more complex reflects an adaptive mechanism allowing females to assess competition and make optimal decisions. The interaction between dose and chemical composition acts as a modulator of *P. polytes*’ behavioral responses to volatiles, too. These findings provide insights into host selection mechanisms in butterflies and offer practical implications for population management.

## Figures and Tables

**Table 1:** VOC profile of *P. polytes* larval frass from *C. limon*-fed Larvae.

No.	Retention Time	Common Name	CAS Registry Number	Category	Relative Abundance (%)
1	111.231	(R)-(+)-Limonene	5989-27-5	Monoterpene Hydrocarbons	1.38 ± 0.38
2	12.158	Linalool	78-70-6	Monoterpenoid Alcohol	1.00 ± 0.37
3	12.490	Perillaldehyde Tetrahydropyran Derivative	3033-23-6	Monoterpenoid Aldehyde Derivative	0.64 ± 0.22
4	13.748	(R)-(+)-Citronellal	2385-77-5	Monoterpenoid Aldehyde	6.06 ± 3.67
5	15.876	Citronellol	106-22-9	Monoterpenoid Alcohol	7.81 ± 2.12
6	16.372	2,3-Dihydrobenzofuran	496-16-2	Aromatic Compound	0.38 ± 0.06
7	16.562	Geraniol	106-24-1	Monoterpenoid Alcohol	0.49 ± 0.21
8	19.232	Geranyl acetate	150-84-5	Monoterpenoid Ester	0.23 ± 0.05
9	19.374	Eugenol	97-53-0	Aromatic Compound	0.34 ± 0.09
10	20.970	Eldanolide	92843-42-0	Lactone	0.59 ± 0.10
11	21.214	α-Ionone	127-41-3	Monoterpenoid Ketone	0.29 ± 0.09
12	21.246	α-Terpineol	515-13-9	Monoterpenoid Alcohol	1.02 ± 0.12
13	22.602	β-Caryophyllene	87-44-5	Sesquiterpene Hydrocarbon	19.70 ± 3.99
14	22.675	β-Ionone	79-77-6	Monoterpenoid Ketone	0.77 ± 0.12
15	22.881	(E)-β-farnesene	18794-84-8	Sesquiterpene Hydrocarbon	25.84 ± 6.04
16	23.601	Olivetol	500-66-3	Aromatic Compound	0.23 ± 0.04
17	23.776	(+)-Nootkatone	17092-92-1	Sesquiterpenoid Ketone	0.41 ± 0.06
18	24.170	β-Bisabolene	495-61-4	Sesquiterpene Hydrocarbon	1.86 ± 0.12
19	25.681	Caryophyllene-related Oxide	19888-34-7	Sesquiterpenoid Oxide	0.34 ± 0.03
20	25.930	Spathulenol	6750-60-3	Sesquiterpenoid Alcohol	2.13 ± 0.27
21	25.993	α-Bergamotol	88034-74-6	Sesquiterpenoid Alcohol	0.25 ± 0.01
22	26.079	Caryophyllene oxide	1139-30-6	Sesquiterpenoid Oxide	1.42 ± 0.57
23	26.086	Isospathulenol	88395-46-4	Sesquiterpenoid Alcohol	0.33 ± 0.07
24	26.238	10,10-Dimethyl-2,6-dimethylenebicyclo [7.2.0] undecan-5-ol	19431-80-2	Monoterpenoid Alcohol	0.54 ± 0.37
25	26.351	Tau-Cadinol	5937-11-01	Sesquiterpenoid Alcohol	0.29 ± 0.04
26	26.891	(1R,7S,E)-7-Isopropyl-4,10-dimethylenecyclodec-5-enol	81968-62-9	Sesquiterpenoid Alcohol	0.27 ± 0.02

**Table 2:** Results of the GLM analyzing the effects of sex, chemical composition, dose, and their interactions on EAG response magnitudes.

Source of Variation	df	Deviance	Mean Deviance	F Value	*p* Value
sex	1	0.6493	0.6493	792.84	<0.001
chemical composition	13	5.2399	0.4031	492.21	<0.001
dose	4	3.4338	0.8585	1048.29	<0.001
sex × chemical composition	13	0.2790	0.0215	26.20	<0.001
sex × dose	4	0.1226	0.0307	37.44	<0.001
chemical composition × dose	52	3.1469	0.0605	73.90	<0.001
sex ×chemical composition × dose	52	0.5837	0.0112	13.71	<0.001

Note: df, degrees of freedom; Deviance, deviance statistic; Mean Deviance, mean deviance; F value, F-statistic; *p* value, significance level.

**Table 3:** Results of the GLM analyzing the effects of chemical composition, sex, dose, and their interactions on behavioral choice in the Y-tube assay.

Source of Variation	df	Deviance	*p* Value (χ^2^)
chemical composition	13	103.089	<0.001
sex	1	3.224	0.073
dose	2	1.430	0.489
chemical composition × sex	13	4.998	0.975
chemical composition × dose	26	54.671	<0.001
sex × dose	2	1.624	0.444
chemical composition × sex × dose	26	23.557	0.601

## Data Availability

The data presented in this study has been uploaded to Mendeley Data and are now openly available at: https://doi.org/10.17632/spj48w8gkj.5.

## Supplementary Materials

The following supporting information can be downloaded at: https://www.mdpi.com/article/10.3390/insects17050452/s1, Supplementary Figure S1: Individual EAG dose–response curves for male *P. polytes* to each compound or mixture. Supplementary Figure S2: Individual EAG dose–response curves for female *P. polytes* to each compound or mixture. Supplementary Table S1: EMM Describing Main Effects on EAG Responses. Supplementary Table S2: EMM comparing the effect of sex on EAG response magnitudes within each chemical stimulus pooled across all doses. Supplementary Table S3: EMM comparing the effect of chemical composition on EAG response magnitudes within each sex pooled across all doses. Supplementary Table S4: EMM for the effect of dose on EAG response magnitudes within specific sex and compound combinations. Supplementary Table S5: EMM (on the logit scale) comparing behavioral choice responses to different chemical stimuli within each sex pooled across all doses.

## Abbreviations

The following abbreviations are used in this manuscript:

CAR	carboxen
CLD	compact letter display
DVB	divinylbenzene
EAG	electroantennography
EMM	estimated marginal means
GC-MS	gas chromatograph–mass spectrometer
GLM	Generalized linear model
HS-SPME	Headspace solid-phase microextraction
PDMS	polydimethylsiloxane
SPME	solid phase microextraction
VOCs	volatile organic compounds
