# Supplementary material for: From Attraction to Repellency: The Olfactory Response Pattern of Papilio polytes to Shared Volatiles from Frass and Host Plants Driven by Chemical Composition
Source: Insects. 2026 Apr 24;17(5):452. doi: 10.3390/insects17050452 (PMC13207816; doi:10.3390/insects17050452)
Supplement: Supplementary file 1 [file insects-17-00452-s001.zip › insects-4128169-supplementary.pdf]

From Attraction to Repellency: The Olfactory Response Pattern of *Papilio polytes* to Shared Volatiles from Frass and Host Plants Driven by Chemical Composition

Xue Wu [1,2], Zengxin Chen [2], Yaqi Yang[2], Huaijian Liao[2], Yunwei Ju [1,*] and Chufei Tang [2,*]

[1] College of Forestry and Grassland, Co-Innovation Center for Sustainable Forestry in Southern China, Nanjing Forestry University, Nanjing 210037, China
[2] Institute of Leisure Agriculture, Jiangsu Academy of Agricultural Sciences, Nanjing 210014, China
* Correspondence: jyw6808@njfu.edu.cn (Ju, Y.); tcf0816@126.com (Tang, C.)

Supplementary Materials

Figure S1. Estimated Marginal Means (EMM) Describing Main Effects on EAG Responses

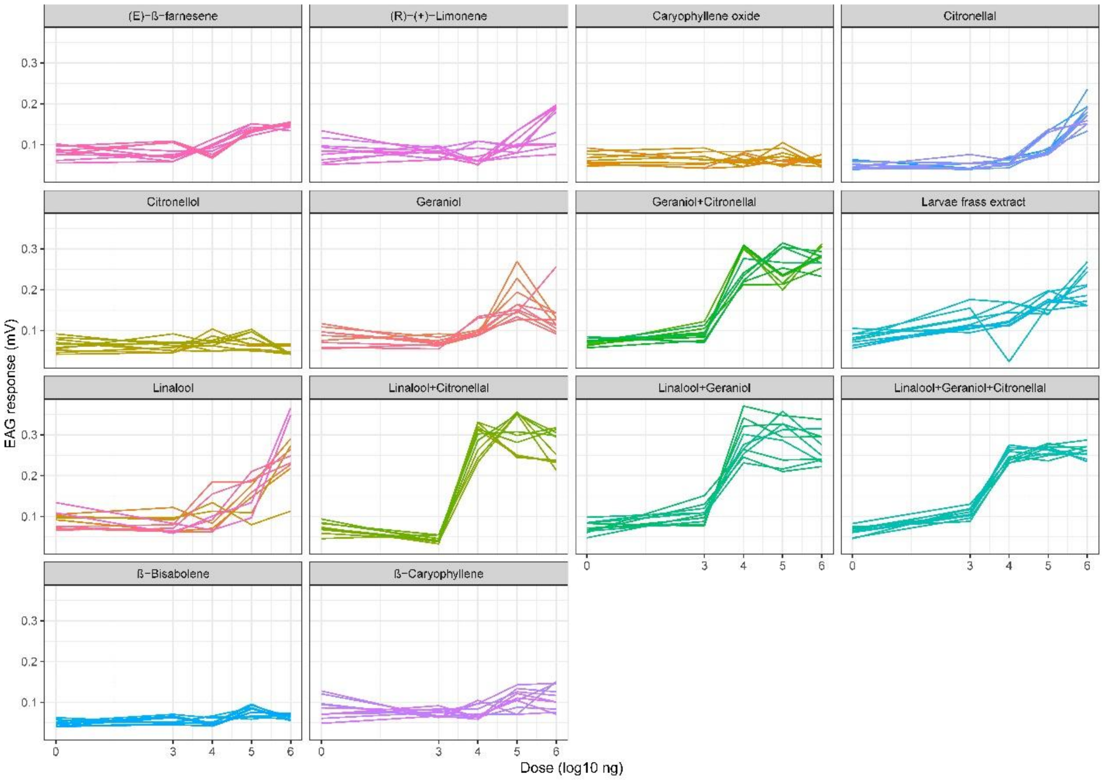

Individual EAG responses of male *P. polytes* to serial dilutions (0, 10^3, 10^4, 10^5, 10^6 ng) of each test compound or mixture. Each colored line represents the response of a single male antenna. The x-axis shows log-transformed dose (log10 ng) to include the solvent control at 0 ng). The y-axis is the EAG response magnitude (mV). Data are presented without

subtraction of the control. Plots are faceted by stimulus (compound or mixture). This figure illustrates the variability among individual male antennae and serves as a supplement to the summary dose–response curves shown in the main text (Figure 1). No statistical comparisons are embedded here; the purpose is visual illustration of raw individual responses.

Figure S2. Estimated Marginal Means (EMM) Describing Main Effects on EAG Responses

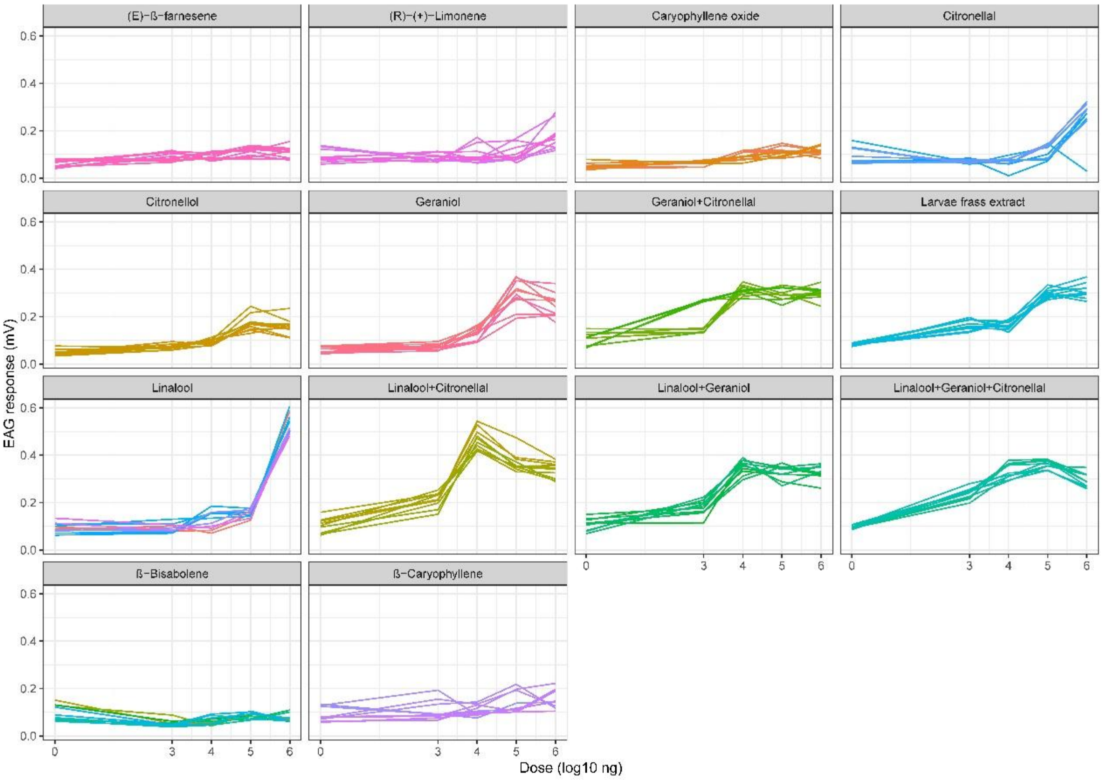

Individual EAG responses of female *P. polytes* to serial dilutions (0, 10^3, 10^4, 10^5, 10^6 ng) of each test compound or mixture. Each colored line represents the response of a single male antenna. The x-axis shows log-transformed dose (log10 ng) to include the solvent control at 0 ng). The y-axis is the EAG response magnitude (mV). Data are presented without subtraction of the control. Plots are faceted by stimulus (compound or mixture). This figure illustrates the variability among individual male antennae and serves as a supplement to the summary dose–response curves shown in the main text (Figure 1). As with the male counterpart, this figure is intended for visual illustration only and does not include embedded statistical comparisons.

Table S1. Estimated Marginal Means (EMM) Describing Main Effects on EAG Responses

Table S1a. EMM of EAG response magnitudes for the effect of sex in adult *P. polytes* pooled across all compounds and doses.

Sex	EMM ± SEM (mV)	95% CI
female	0.167 ± 0.001 a	0.165–0.169
male	0.124 ± 0.001 b	0.122–0.126

Note: Values are expressed as EMM ± SEM. CI represents the 95% symmetric confidence interval calculated using the emmeans method. Letter markings indicate significance based on Tukey HSD correction (α= 0.05), where different letters denote significant differences between sexes.

Table S1b. EMM of EAG response magnitudes for the effect of chemical composition in adult *P. polytes* pooled across all sexes and doses.

Compound	EMM ± SEM (mV)	95% CI
Linalool+Citronellal	0.247 ± 0.003 a	0.241–0.253
Linalool+Geraniol	0.232 ± 0.003 b	0.226–0.238
Linalool+Geraniol+Citronellal	0.226 ± 0.003 bc	0.220–0.232
Geraniol+Citronellal	0.216 ± 0.003 c	0.210–0.222
Larvae frass extract	0.171 ± 0.003 d	0.165–0.177
Linalool	0.169 ± 0.003 d	0.163–0.175
Geraniol	0.137 ± 0.003 e	0.131–0.143
β-Caryophyllene	0.105 ± 0.003 f	0.099–0.111
(R)-(+)-Limonene	0.104 ± 0.003 f	0.098–0.110
Citronellal	0.103 ± 0.003 f	0.097–0.109
(E)-β-farnesene	0.100 ± 0.003 f	0.094–0.106
Citronellol	0.087 ± 0.003 g	0.081–0.093
Caryophylleneoxide	0.075 ± 0.003 gh	0.069–0.081
β-Bisabolene	0.068 ± 0.003 h	0.062–0.074

Note: Values are expressed as EMM ± SEM. Letter markings indicate significance based on Tukey HSD correction (α= 0.05), where different letters denote significant differences among chemical stimuli.

Table S1c. EMM of EAG response magnitudes for the effect of dose in adult *P. polytes* pooled across all sexes and compounds.

Dose (ng)	EMM ± SEM (mV)	95% CI
1×10^6	0.208 ± 0.002 a	0.204–0.212
1×10^5	0.186 ± 0.002 b	0.182–0.190
1×10^4	0.157 ± 0.002 c	0.153–0.161
1×10^3	0.100 ± 0.002 d	0.096–0.104
0	0.079 ± 0.002 e	0.075–0.083

Note: Values are expressed as EMM ± SEM. Letter markings indicate significance based on Tukey HSD correction (α= 0.05), where different letters denote significant differences among dose levels.

Table S2. EMM comparing the effect of sex on EAG response magnitudes within each chemical stimulus pooled across all doses.

Compound	Sex	EMM ± SEM (mV)	95% CI
(R)-(+)-Limonene	female	0.112 ± 0.004 a	0.104–0.120
	male	0.097 ± 0.004 b	0.089–0.105
Caryophylleneoxide	female	0.086 ± 0.004 a	0.078–0.094
	male	0.064 ± 0.004 b	0.056–0.072
Citronellal	female	0.121 ± 0.004 a	0.113–0.129
	male	0.085 ± 0.004 b	0.077–0.093
Citronellol	female	0.109 ± 0.004 a	0.101–0.117
	male	0.064 ± 0.004 b	0.056–0.072
Geraniol	female	0.161 ± 0.004 a	0.153–0.169
	male	0.112 ± 0.004 b	0.104–0.120
Geraniol+Citronellal	female	0.241 ± 0.004 a	0.233–0.249
	male	0.192 ± 0.004 b	0.184–0.200
Larvae frass extract	female	0.204 ± 0.004 a	0.196–0.212
	male	0.139 ± 0.004 b	0.131–0.147
Linalool	female	0.201 ± 0.004 a	0.193–0.209
	male	0.137 ± 0.004 b	0.129–0.145
Linalool+Citronellal	female	0.298 ± 0.004 a	0.290–0.306
	male	0.196 ± 0.004 b	0.188–0.204
Linalool+Geraniol	female	0.259 ± 0.004 a	0.251–0.267
	male	0.205 ± 0.004 b	0.197–0.213
Linalool+Geraniol+Citronellal	female	0.264 ± 0.004 a	0.256–0.272
	male	0.188 ± 0.004 b	0.180–0.196
β-Bisabolene	female	0.074 ± 0.004 a	0.066–0.082
	male	0.061 ± 0.004 b	0.053–0.069
β-Caryophyllene	female	0.119 ± 0.004 a	0.111–0.127
	male	0.091 ± 0.004 b	0.083–0.099

Note: Values are expressed as EMM ± SEM. Letter markings are used to compare differences between sexes within each compound based on Tukey HSD correction (α= 0.05).

Table S3. EMM comparing the effect of chemical composition on EAG response magnitudes within each sex pooled across all doses.

Compound	Female		Male	
	EMM ± SEM (mV)	95% CI	EMM ± SEM (mV)	95% CI
Linalool+Citronellal	0.298 ± 0.004 a	0.290–0.306	0.196 ± 0.004 a	0.188–0.204
Linalool+Geraniol+Citronellal	0.264 ± 0.004 b	0.256–0.272	0.188 ± 0.004 a	0.180–0.196
Linalool+Geraniol	0.259 ± 0.004 bc	0.251–0.267	0.205 ± 0.004 a	0.197–0.213
Geraniol+Citronellal	0.241 ± 0.004 c	0.233–0.249	0.192 ± 0.004 a	0.184–0.200
Larvae frass extract	0.204 ± 0.004 d	0.196–0.212	0.139 ± 0.004 b	0.131–0.147
Linalool	0.201 ± 0.004 d	0.193–0.209	0.137 ± 0.004 b	0.129–0.145
Geraniol	0.161 ± 0.004 e	0.153–0.169	0.112 ± 0.004 c	0.104–0.120
(E)-β-farnesene	0.093 ± 0.004 gh	0.085–0.101	0.107 ± 0.004 cd	0.099–0.115
(R)-(+)-Limonene	0.112 ± 0.004 fg	0.104–0.120	0.097 ± 0.004 cde	0.089–0.105
β-Caryophyllene	0.119 ± 0.004 f	0.111–0.127	0.091 ± 0.004 de	0.083–0.099
Citronellal	0.121 ± 0.004 f	0.113–0.129	0.085 ± 0.004 e	0.077–0.093
Citronellol	0.109 ± 0.004 fg	0.101–0.117	0.064 ± 0.004 f	0.056–0.072
Caryophylleneoxide	0.086 ± 0.004 h	0.078–0.094	0.064 ± 0.004 f	0.056–0.072
β-Bisabolene	0.074 ± 0.004 h	0.066–0.082	0.061 ± 0.004 f	0.053–0.069

Note: Values are expressed as EMM ± SEM. Letter markings are used for pairwise comparisons among compounds within the same sex based on Tukey HSD correction (α= 0.05).

Table S4. EMM for the effect of dose on EAG response magnitudes within specific sex and compound combinations.

Compound	Sex	Dose (ng)	EMM ± SEM (mV)	95% CI
(E)-β-farnesene	female	1×10^6	0.109 ± 0.009 a	0.091–0.127
		1×10^5	0.114 ± 0.009 a	0.096–0.132
		1×10^4	0.093 ± 0.009 a	0.075–0.111
		1×10^3	0.091 ± 0.009 ab	0.073–0.109
		0	0.059 ± 0.009 b	0.042–0.076
	male	1×10^6	0.147 ± 0.009 a	0.129–0.165
		1×10^5	0.138 ± 0.009 a	0.120–0.156
		1×10^4	0.090 ± 0.009 b	0.072–0.108
		1×10^3	0.081 ± 0.009 b	0.063–0.099
		0	0.080 ± 0.009 b	0.063–0.097
(R)-(+)-Limonene	female	1×10^6	0.177 ± 0.009 a	0.159–0.195
		1×10^5	0.103 ± 0.009 b	0.085–0.121
		1×10^4	0.097 ± 0.009 b	0.079–0.115
		1×10^3	0.088 ± 0.009 b	0.070–0.106
		0	0.095 ± 0.010 b	0.076–0.114
	male	1×10^6	0.146 ± 0.009 a	0.128–0.164
		1×10^5	0.100 ± 0.009 b	0.082–0.118
		1×10^4	0.072 ± 0.009 b	0.054–0.090
		1×10^3	0.081 ± 0.009 b	0.063–0.099
		0	0.086 ± 0.010 b	0.067–0.105
Caryophylleneoxide	female	1×10^6	0.113 ± 0.009 a	0.095–0.131
		1×10^5	0.111 ± 0.009 a	0.093–0.129
		1×10^4	0.092 ± 0.009 ab	0.074–0.110
		1×10^3	0.064 ± 0.009 bc	0.046–0.082
		0	0.051 ± 0.010 c	0.032–0.070
	male	1×10^6	0.058 ± 0.009 a	0.040–0.076
		1×10^5	0.069 ± 0.009 a	0.051–0.087
		1×10^4	0.063 ± 0.009 a	0.045–0.081
		1×10^3	0.063 ± 0.009 a	0.045–0.081
		0	0.066 ± 0.010 a	0.047–0.085
Citronellal	female	1×10^6	0.259 ± 0.009 a	0.241–0.277
		1×10^5	0.111 ± 0.009 b	0.093–0.129
		1×10^4	0.068 ± 0.009 c	0.050–0.086
		1×10^3	0.069 ± 0.009 c	0.051–0.087
		0	0.097 ± 0.009 bc	0.079–0.115
	male	1×10^6	0.174 ± 0.009 a	0.156–0.192
		1×10^5	0.098 ± 0.009 b	0.080–0.116

Compound	Sex	Dose (ng)	EMM ± SEM (mV)	95% CI
		1×10^4	0.057 ± 0.009 c	0.039–0.075
		1×10^3	0.049 ± 0.009 c	0.031–0.067
		0	0.049 ± 0.009 c	0.031–0.067
Citronellol	female	1×10^6	0.158 ± 0.009 a	0.140–0.176
		1×10^5	0.173 ± 0.009 a	0.155–0.191
		1×10^4	0.093 ± 0.009 b	0.075–0.111
		1×10^3	0.073 ± 0.009 bc	0.055–0.091
		0	0.048 ± 0.009 c	0.031–0.065
	male	1×10^6	0.050 ± 0.009 a	0.032–0.068
		1×10^5	0.073 ± 0.009 a	0.055–0.091
		1×10^4	0.071 ± 0.009 a	0.053–0.089
		1×10^3	0.062 ± 0.009 a	0.044–0.080
		0	0.065 ± 0.009 a	0.048–0.082
Geraniol	female	1×10^6	0.250 ± 0.009 b	0.232–0.268
		1×10^5	0.297 ± 0.009 a	0.279–0.315
		1×10^4	0.128 ± 0.009 c	0.110–0.146
		1×10^3	0.074 ± 0.009 d	0.056–0.092
		0	0.059 ± 0.009 d	0.041–0.077
	male	1×10^6	0.132 ± 0.009 b	0.114–0.150
		1×10^5	0.172 ± 0.009 a	0.154–0.190
		1×10^4	0.100 ± 0.009 bc	0.082–0.118
		1×10^3	0.071 ± 0.009 c	0.053–0.089
		0	0.084 ± 0.009 c	0.066–0.102
Geraniol+Citronellal	female	1×10^6	0.301 ± 0.009 a	0.283–0.319
		1×10^5	0.296 ± 0.009 a	0.278–0.314
		1×10^4	0.310 ± 0.009 a	0.292–0.328
		1×10^3	0.191 ± 0.009 b	0.173–0.209
		0	0.107 ± 0.009 c	0.089–0.125
	male	1×10^6	0.277 ± 0.009 a	0.259–0.295
		1×10^5	0.254 ± 0.009 a	0.236–0.272
		1×10^4	0.262 ± 0.009 a	0.244–0.280
		1×10^3	0.094 ± 0.009 b	0.076–0.112
		0	0.071 ± 0.009 b	0.053–0.089
Larvae frass extract	female	1×10^6	0.309 ± 0.009 a	0.291–0.327
		1×10^5	0.299 ± 0.009 a	0.281–0.317
		1×10^4	0.164 ± 0.009 b	0.146–0.182
		1×10^3	0.167 ± 0.009 b	0.149–0.185
		0	0.081 ± 0.009 c	0.063–0.099
	male	1×10^6	0.203 ± 0.009 a	0.185–0.221

Compound	Sex	Dose (ng)	EMM ± SEM (mV)	95% CI
		1×10^{5}	0.167 ± 0.009 b	0.149–0.185
		1×10^{4}	0.125 ± 0.009 c	0.107–0.143
		1×10^{3}	0.123 ± 0.009 c	0.105–0.141
		0	0.078 ± 0.009 d	0.060–0.096
		1×10^{6}	0.538 ± 0.009 a	0.520–0.556
		1×10^{5}	0.155 ± 0.009 b	0.137–0.173
	female	1×10^{4}	0.125 ± 0.009 bc	0.107–0.143
		1×10^{3}	0.093 ± 0.009 c	0.075–0.111
Linalool		0	0.094 ± 0.009 c	0.076–0.112
		1×10^{6}	0.258 ± 0.009 a	0.240–0.276
		1×10^{5}	0.149 ± 0.009 b	0.131–0.167
	male	1×10^{4}	0.106 ± 0.009 c	0.088–0.124
		1×10^{3}	0.080 ± 0.009 c	0.062–0.098
		0	0.093 ± 0.009 c	0.075–0.111
		1×10^{6}	0.337 ± 0.009 b	0.319–0.355
		1×10^{5}	0.370 ± 0.009 b	0.352–0.388
	female	1×10^{4}	0.469 ± 0.009 a	0.451–0.487
		1×10^{3}	0.211 ± 0.009 c	0.193–0.229
		0	0.103 ± 0.009 d	0.085–0.121
Linalool+Citronellal		1×10^{6}	0.270 ± 0.009 a	0.252–0.288
		1×10^{5}	0.304 ± 0.009 a	0.286–0.322
	male	1×10^{4}	0.293 ± 0.009 a	0.275–0.311
		1×10^{3}	0.043 ± 0.009 b	0.025–0.061
		0	0.072 ± 0.009 b	0.054–0.090
		1×10^{6}	0.329 ± 0.009 a	0.311–0.347
		1×10^{5}	0.327 ± 0.009 a	0.309–0.345
	female	1×10^{4}	0.352 ± 0.009 a	0.334–0.370
		1×10^{3}	0.179 ± 0.009 b	0.161–0.197
		0	0.108 ± 0.009 c	0.090–0.126
Linalool+Geraniol		1×10^{6}	0.270 ± 0.009 a	0.252–0.288
		1×10^{5}	0.291 ± 0.009 a	0.273–0.309
	male	1×10^{4}	0.287 ± 0.009 a	0.269–0.305
		1×10^{3}	0.107 ± 0.009 b	0.089–0.125
		0	0.073 ± 0.009 b	0.055–0.091
		1×10^{6}	0.293 ± 0.009 b	0.275–0.311
		1×10^{5}	0.360 ± 0.009 a	0.342–0.378
Linalool+Geraniol+Citronellal	female	1×10^{4}	0.332 ± 0.009 a	0.314–0.350
		1×10^{3}	0.238 ± 0.009 c	0.220–0.256
		0	0.095 ± 0.009 d	0.077–0.113

Compound	Sex	Dose (ng)	EMM ± SEM (mV)	95% CI
	male	1×10^6	0.258 ± 0.009 a	0.240–0.276
		1×10^5	0.261 ± 0.009 a	0.243–0.279
		1×10^4	0.249 ± 0.009 a	0.231–0.267
		1×10^3	0.109 ± 0.009 b	0.091–0.127
		0	0.064 ± 0.009 c	0.046–0.082
β-Bisabolene	female	1×10^6	0.074 ± 0.009 ab	0.056–0.092
		1×10^5	0.086 ± 0.009 ab	0.068–0.104
		1×10^4	0.064 ± 0.009 ab	0.046–0.082
		1×10^3	0.052 ± 0.009 b	0.034–0.070
		0	0.096 ± 0.009 a	0.078–0.114
	male	1×10^6	0.064 ± 0.009 a	0.046–0.082
		1×10^5	0.079 ± 0.009 a	0.061–0.097
		1×10^4	0.053 ± 0.009 a	0.035–0.071
		1×10^3	0.058 ± 0.009 a	0.040–0.076
		0	0.049 ± 0.009 a	0.031–0.067
β-Caryophyllene	female	1×10^6	0.154 ± 0.009 a	0.136–0.172
		1×10^5	0.140 ± 0.009 ab	0.122–0.158
		1×10^4	0.108 ± 0.009 bc	0.090–0.126
		1×10^3	0.107 ± 0.009 bc	0.089–0.125
		0	0.089 ± 0.009 c	0.071–0.107
	male	1×10^6	0.112 ± 0.009 a	0.094–0.130
		1×10^5	0.108 ± 0.009 ab	0.090–0.126
		1×10^4	0.077 ± 0.009 ab	0.059–0.095
		1×10^3	0.073 ± 0.009 b	0.055–0.091
		0	0.085 ± 0.009 ab	0.067–0.103

Note: Values are expressed as EMM ± SEM. Letter markings are used for comparisons among different doses within each specific sex and compound combination based on Tukey HSD correction (α= 0.05).

Table S5. EMM (on the logit scale) comparing behavioral choice responses to different chemical stimuli within each sex pooled across all doses.

Sex	Compound	EMM (logit)	SE	Lower CL	Upper CL	Group
Female	Linalool+Geraniol+Citronellal	-11.809	0.295	-175.964	-0.602	a
	Larvae frass extract	-0.769	0.276	-130.867	-0.228	ab
	β-Caryophyllene	-0.161	0.268	-0.686	0.364	abc
	(E)-β-farnesene	-0.087	0.258	-0.593	0.419	abc
	Citronellal	0.039	0.302	-0.552	0.631	abc
	Linalool+Citronellal	0.136	0.267	-0.387	0.660	abc
	(R)-(+)-Limonene	0.202	0.260	-0.308	0.712	bc
	Linalool	0.361	0.290	-0.207	0.930	bc
	Citronellol	0.366	0.272	-0.167	0.899	bc
	Linalool+Geraniol	0.682	0.288	0.117	1.247	c
	Caryophylleneoxide	0.849	0.305	0.251	1.446	c
	Geraniol+Citronellal	0.880	0.283	0.326	1.434	c
	Geraniol	0.897	0.309	0.292	1.502	c
	β-Bisabolene	0.897	0.309	0.292	1.502	c
Male	Linalool+Geraniol+Citronellal	-0.996	0.316	-161.645	-0.376	a
	Larvae frass extract	-0.642	0.264	-115.949	-0.124	ab
	β-Caryophyllene	-0.372	0.259	-0.879	0.135	abc
	(E)-β-farnesene	-0.318	0.272	-0.851	0.216	abc
	Citronellol	-0.288	0.282	-0.840	0.264	abc
	Citronellal	0.035	0.253	-0.461	0.532	abc
	Linalool	0.105	0.265	-0.415	0.625	abc
	(R)-(+)-Limonene	0.138	0.267	-0.385	0.662	abc
	Linalool+Geraniol	0.287	0.252	-0.207	0.780	abc
	Linalool+Citronellal	0.292	0.289	-0.276	0.859	abc
	Geraniol+Citronellal	0.396	0.256	-0.105	0.897	bc
	β-Bisabolene	0.546	0.277	0.002	1.090	bc
	Geraniol	0.546	0.277	0.002	1.090	bc
	Caryophylleneoxide	0.687	0.270	0.159	1.216	c

Note: Values are expressed as EMM ± SE on the logit scale. CL represents the confidence limits calculated from the binomial GLM. Letter markings denote significant differences among compounds within the same sex based on Tukey adjustment ($p > 0.05$).
